# Exploring Ethical, Ecological, and Health Factors Influencing the Acceptance of Cultured Meat among Generation Y and Generation Z

**DOI:** 10.3390/nu15132935

**Published:** 2023-06-28

**Authors:** Lucie Pilařová, Tereza Balcarová, Ladislav Pilař, Lucie Kvasničková Stanislavská, Joanna Rosak-Szyrocka, Jana Pitrová, Pavel Moulis, Roman Kvasnička

**Affiliations:** 1 Department of Management, Faculty of Economics and Management, Czech University of Life Sciences Prague, 165 21 Prague, Czech Republickvasnickova@pef.czu.cz (L.K.S.);; 2 Department of Production Engineering and Safety, Faculty of Management, Czestochowa University of Technology, 42-201 Częstochowa, Poland; 3 Department of Systems Engineering, Faculty of Economics and Management, Czech University of Life Sciences Prague, 165 21 Prague, Czech Republic

**Keywords:** cultured meat, generation Z, generation Y, ethical awareness, ecological awareness, nutrition benefits

## Abstract

Growing research and technological development is making the commercial production of cultured meat as a sustainable alternative to livestock-derived meat an increasing reality. However, to competitively position cultured meat on the food market, appropriate marketing and communication tailored to specific demographics is required. We aimed to define the motives that influence the willingness to include cultured meat in consumption based on age, specifically in Generation Z and Generation Y. To achieve this, data from a questionnaire survey that asked about ethical, ecological and health and safety factors around cultured meat was collected from 740 respondents (301 Generation Z and 439 Generation Y) and analyzed using the Mann–Whitney test and structural equation modeling. Generation Z were significantly more likely than Generation Y (*p* < 0.05) to consider cultured meat healthier than conventional meat because of the possibility of adjusting the composition and nutrient content. Generation Z were also significantly less concerned than Generation Y (*p* < 0.05) about the consequences that consuming cultured meat might have on human health. In Generation Z, ethical, ecological and health and safety factors significantly influenced their willingness to consume cultured meat (all *p* < 0.01). In conclusion, we confirmed the influence of ecological and ethical awareness, as well as health and safety, on willingness to include cultured meat in consumption; these areas could be targeted when marketing cultured meat.

## 1. Introduction

The potential idea of adopting cultured meat as an alternative source of protein turned into real intentions of partially replacing traditional livestock meat with protein alternatives, including cultured meat [1,2]. As a result, a number of studies have focused on acceptance of cultured meat by potential consumers, together with their concerns around this novel food source [3,4,5,6,7,8]. One reason for the increasing interest and investment in cultured meat is climate change, with livestock production being a major contributor to carbon emissions [9,10,11]; however, the world’s meat consumption continues to increase as the population grows [2,11,12]. Although plant-based alternatives to meat have been developed, studies show that these products cannot replace the mineral, vitamin and amino acid content of meat [11,13,14,15,16,17]. By contrast, cultured meat may provide a nutritionally similar, sustainable alternative to traditional meat.

Although cultured meat is not widely available, scientific studies are already revealing the drivers of potential market adoption, including modeling the first customers who are willing to accept and adopt cultured meat into their diet [9,18,19]. A number of barriers have also been identified, primarily the fear and reluctance to eat new foods, called neophobia [9,15,17,20]. However, differences in the adoption of cultured meat among different younger generations who are the most likely potential adopters of cultured meat are not known [17,21,22,23,24,25].

### 1.1. Theoretical Background

Meat culturing in a laboratory has been proposed as an alternative to current livestock-based meat production systems [26,27,28,29]. The growing interest in cultured meat reflects global concerns about how best to satisfy the nutritional needs of humans while mitigating the effect of livestock production on the environment [30,31,32]; although meat production significantly contributes to rising greenhouse gas emissions, meat is an essential component in the human diet [33,34,35]. As a result, interest has turned to sourcing a high-quality, nutrient-dense, environmentally friendly meat alternative, with cultured meat being an attractive solution [4,6,7,8,36,37].

Cultured meat is referred to using a number of different terms, including lab-grown meat, artificial meat, cell-based meat, cell-cultured meat, slaughter-free meat, synthetic meat, clean meat, and in vitro meat [38,39,40,41]. For the purposes of this paper, the term “cultured meat” is primarily used, drawing on research from Reis et al. (2020) [42], who found that terms emphasizing the process of developing such meat products (including “cultivated”, “cultured”, and “cell-cultured”) were generally viewed as more transparent and therefore more favorable by the public. Terms emphasizing the artificial, manufactured nature of the product (including “synthetic”, “lab-grown”, and “in vitro”) were less desirable. Cultured meat is developed in a laboratory setting via the following steps: (i) collection of a biopsy of animal cells, usually from a living animal; (ii) use of cells to create a “cell line”, which the National Cancer Institute (2022) [43] describes as a new group of cells adapted for continuous future growth; (iii) growing these cells and differentiating them into the desired tissue type; and (iv) using physical (typically electrical) as well as biochemical stimuli to further process these cells into a product that resembles traditional meat in appearance, texture, nutrient content and taste [13,16,44].

### 1.2. Attitudes toward Cultured Meat

Attitudes toward cultured meat significantly impact the likelihood of its adoption by consumers. Many consumers are open to trying cultured meat; however, Valente et al. (2019) [29] found that individual opinions and attitudes affect the likelihood of consuming cultured meat regularly, or replacing traditionally raised meat with cultured meat. Chriki et al. (2021) [45] found that consumer attitudes were heavily influenced by how “high-tech” they perceived a certain product to be, especially when that product could be associated with food. Siddiqui et al. (2022) [11] found similar results, stating that participants tended to view food technology as more favorable when it was used to develop plant-based foods rather than those containing animal proteins and byproducts.

Consumer attitudes toward cultured meat can also be affected by ethical considerations [3]. For example, research shows that many consumers struggle to conceive of cultured meat as a net positive for humanity because they believe it is “unnatural” and therefore unethical, with Verbeke et al. (2015) [20] finding that consumers argued cultured meat was “against nature”. Participants in a study conducted by Bryant and Barnett (2020) [22] went a step further, stating that processing meat in a lab was a form of “playing God”. In addition, Jetzke et al. (2016) [46] stated that the role of animals within a complex ecosystem changes when meat is lab grown and humans lose touch with the origin of animal food. By contrast, cultured meat may have positive ethical connotations when viewed as a means to reduce livestock production and animal slaughter [47,48].

However, concerns around the ethics of cultured meat go beyond its perception as artificial [22]. Bryant and Barnett (2020) [22] found that consumers expressed anxieties around the newness of cultured meat technology, with some concerned that the process was not sufficiently understood and that it could have unforeseen negative consequences, both for consumers and the planet as a whole. Fear of cultured meat was also linked to distrust of government oversight bodies and public regulation systems, highlighting the importance of transparency and open communication around technological development, particularly when involving food products [49,50].

Another influence on attitudes towards cultured meat consumption is its impact at a societal level, which can be both positive and negative, according to Bryant and Barnett (2020) [22]. They found that positive attitudes included recognizing the potential of cultured meat to use fewer resources and to produce more affordable and accessible high-protein food for lower-income and food-insecure populations. However, the most common positive attitude towards adopting cultured meat focused on the technology’s ability to dramatically improve conditions for livestock animals [29]. Negative attitudes included concerns about the impact of widespread, laboratory-based meat production on job security for people currently making their living via farm-based livestock rearing [22].

Consumer attitudes toward consumption of cultured meat are also significantly influenced by perceptions and expectations around two key product attributes: taste and price. Research indicates that some consumers rate taste (including texture) as a fundamental factor in engaging with a new food product [51]. Consumers are also skeptical about the price point of cultured meat, possibly because of a mental association between cutting-edge technology and higher prices [22], despite research estimating that by 2035, certain plant-based substitutes and cultured meats will be five times cheaper than the existing animal proteins [28]. Liu et al. (2021) [52] found that consumers who were willing to try cultured meat were generally less willing to pay a higher price for it than traditional meat, with many stating that “artificial meat will never be better than real meat” (p. 21). However, Szejda et al. (2021) [5] found that at least half of consumers labeled themselves as “at least somewhat likely” to pay more for cultured meat. These contrasting results suggest that further research into the impact of price on attitudes towards cultured meat is needed.

### 1.3. Adoption of Cultured Meat

Cultured meat is a relatively new innovation within food technology, and, as with any new product, there are concerns about its acceptance and adoption by the public. According to previous studies, consumers’ main concerns are the ethical aspects of cultured meat consumption [20,22]; transparency of public communication around technological development of cultured meat [49,50]; and the impact of widespread adoption of cultured meat on people making their living through farming [53]. Siddiqui et al. (2022) [11] found a number of barriers to adopting a novel food product in particular, including social norms around the type of food being consumed and goals related to eating, such as consuming food that is considered healthy. An early assessment of adoption likelihood conducted by Verbeke et al. (2015) [20] found that the idea of meat grown in a lab setting elicited strong negative reactions from study participants, which the researchers categorized as “visceral” because they “were driven by emotions as much as by reasoning” (p. 8). Participants expressed hesitation around adoption of cultured meat, with some stating that they wanted to avoid consuming such products during their lifetime. Although they believed this was possible, they also believed cultured meat would become a necessary evil in future society [20], because the planet’s natural resources cannot meet the growing demand for meat [54].

However, adoption likelihood has changed in more recent years, with consumers stating that cultured meat is a technology for the future by 2017 [55]. It is important to note that attitudes can vary between countries. For example, over half of consumers in Germany, Italy, and France indicate that they would find the consumption of cultured meat acceptable; in the U.S., over two-thirds of consumers state that they would be open to at least trying cultured meat, and anywhere between a quarter and half of consumers state that they could see themselves purchasing and consuming cultured meat on a regular basis [5,19,25,56,57,58]. Outside of the U.S. and Europe, researchers in China found that around a third of Chinese consumers are willing to try cultured meat, but that many participants who indicated their willingness to try cultured meat also state that they would not be willing to eat cultured meat regularly or to accept cultured meat as an alternative to traditional meat [59,60].

Research has found a number of country-specific factors that influence consumers’ willingness to try cultured meat. Szejda et al. (2021) [5] found that in the U.S. and UK, consumers were more willing to try cultured meat when they were presented with certain facts, such as that cultured meat is more likely to be free from animal-borne pathogens; that cultured meat does not require antibiotics to grow, which reduces the risk of antibiotic resistance in consumers; and that with fewer resources required, the production of cultured meat could conceivably increase food security and access to meat protein on a global level. In the UK, consumers also considered the environmental benefits over traditional livestock rearing methods in their willingness to try cultured meat. However, current attitudes and the level of cultured meat adoption vary not only geographically, but also based on societal demographics [61,62].

### 1.4. Generational Differences in Cultured Meat Adoption Attitudes

Preliminary research indicates that younger people may be more open both to trying cultured meat and to consuming it on a regular basis [5,22,29,61,62,63]; the likelihood of adopting cultured meat may therefore vary based on the generation with which a consumer identifies. Szejda et al. (2021) [5] analyzed consumer attitudes by generation in a study assessing willingness to try cultured meat, dividing participants into four generations: Baby Boomers, Generation X, Millennials, and Generation Z. The Pew Research Center defines Baby Boomers as those born between 1946 and 1964, Generation X as those born between 1965 and 1980, Millennials as those born between 1981 and 1996, and Generation Z as those born during or after 1997 [64].

Szejda et al. (2021) [5] reported that members of younger generations were more open-minded when it came to consuming cultured meat: “88% of Generation Z and 85% of Millennials” rated themselves as “at least somewhat open” to the prospect of trying cultured meat, compared with “77% of Generation X and 72% of Baby Boomers” (p. 1). Research conducted in Italy also found that belonging to Generation Z was a key characteristic in potential customer profiles for cultured meat [25]. Mancini and Antonioli (2019) [25] hypothesized that this tendency may be due in part to a greater familiarity with cultured meat among younger generations, finding that “familiarity with cultured meat was higher amongst younger generations. Over 10% of Generation Z in both the U.S. and the UK stated that they were very or extremely familiar with the concept, whilst around two-thirds of Baby Boomers were not at all familiar with cultured meat” (p. 9). Familiarity with a food technology is a strong predictor of acceptance, in large part because of the human tendency to reject food products never encountered before [65,66,67].

Research on Chinese consumers has yielded conflicting results regarding the impact of age and generation on a person’s likelihood of or openness to consuming cultured meat. Liu et al. (2021) [52] found that younger Chinese consumers were open to trying cultured meat, but, as mentioned previously, they were unwilling to pay more for cultured meat than they would for traditional meat; in fact, they would only be willing to buy and consume cultured meat if it was sold at a lower price point than traditional meat. Noting research indicating that young people of different nationalities are more open to the prospect of consuming cultured meat [1,19], Liu et al. (2021) [52] hypothesized an effective method of increasing acceptance of cultured meat among young Chinese consumers: to advertise cultured meat as a convenient alternative to traditional meat for making snack foods upon which young Chinese consumers are increasingly reliant due to their busy lifestyles [68].

## 2. Materials and Methods

Given this background, our aim was to define the motives that influence the willingness to include cultured meat in consumption by Generation Z and Generation Y. To achieve this, a survey was conducted using a questionnaire developed from previous research conducted in France in [69], as well as a literature search in the field of contemporary perception of cultured meat and motivating people to switch to veganism, vegetarianism and flexitarianism [70,71,72]. The questionnaire consisted of 13 questions, including 7 questions about ethical and ecological awareness around cultured meat and belief in its health benefits and safety (5-point Likert scale), 1 question focused on willingness to include cultured meat in consumption (5-point Likert scale), and 5 sociodemographic questions (close-ended questions).

As a follow-up to the previous research from France [69], the same method of disseminating the questionnaire was used. The survey was widely distributed on social networks: Facebook, LinkedIn and Twitter. Respondents were invited to spread the questionnaire further among other users on social networks. Given that the questionnaire was translated into Czech, we assumed that all respondents lived in the Czech Republic. The questionnaire was completed by a total of 740 respondents, including 301 individuals from Generation Z and 439 from Generation Y. The sample consisted of 62% women and 48% men.

Defining each generation can vary slightly depending on the source. For this research, we used the following definitions [73]:

- Generation Z, often referred to as “Gen Z” or “iGeneration”, generally consists of individuals born between the mid-1990s and the mid-2010s. For this research, we used the range of 1997–2012 based on a previous definition from [74].
- Generation Y, commonly known as “Millennials”, encompasses individuals born between the early 1980s and the mid-1990s. For this research, we used the range of 1981–1996 based on a previous definition from [75].

The Mann–Whitney U-test and structural equation modeling were utilized to identify differences between generations.

### 2.1. Mann–Whitney U-Test

The Mann–Whitney U-test compares the means of two independent groups to assess whether a statistically significant difference exists between them. This non-parametric test is appropriate for analyzing data that deviate from normality and/or have unequal variances. The Mann–Whitney U-test is especially robust when sample sizes are small or moderate [76]. Statistical significance was accepted when *p* < 0.05.

### 2.2. Structural Equation Modeling (SEM)

SEM is a multivariate analysis method used to explore complex relationships between variables and outcome measures; SEM is sometimes referred to as the second generation of multivariate statistics. This method enables simultaneous analysis of all variables in one model (one or more independent variables in relation to one or more dependent variables). This allows evaluation of a factor comprised of multiple variables without needing to analyze each variable individually, thus providing a single, yet more complex representation of the data [77].

#### 2.2.1. Normality Assessment

Normality assessment can use either visual or statistical methods. Statistical assessment using skewness and kurtosis values, which are the two important components of normality [78], is considered more objective for use in SEM. The characteristics of skewness and kurtosis were preferred in this work over those of the Shapiro–Wilk W test and the Kolmogorov–Smirnov D test due to their lower sensitivity to sample size [79]. A dataset in which skewness and kurtosis is between −2 and 2 is considered to have a normal distribution [80].

#### 2.2.2. Model Representativeness

Confirmatory factor analysis is used to determine how well a particular factor in the model represents the data, and whether application of the prescribed model is appropriate for the data and its structure. This property can be achieved assuming a good “fit” of the model. The most commonly used fit indices are as follows: GFI, goodness-of-fit index [81]; RMSEA, root mean square error of approximation [82]; NFI, normed fit index [83]; TLI, Tucker–Lewis coefficient [83]; CFI, comparative fit index [84]; and IFI, incremental fit index [83].

Values above 0.9 for the GFI, NFI, TLI, CFI, and IFI indices indicate a good fit of the model. RMSEA values greater than 0.1 indicate a poor fit. RMSEA values between 0.08 and 0.1 indicate an average fit, those between 0.05 and 0.08 indicate a good fit, and those less than 0.05 indicate a very good fit of the model.

#### 2.2.3. Evaluation of Hypotheses

Based on a set of working hypotheses, statistical hypotheses (null, “0” and alternative, “A”) were created using a standardized procedure [85]. To confirm the hypotheses based on path analysis using SEM, a minimum correlation value (standardized regression) of 0.3 was required at a significance level of *p* < 0.05. In analyzing the mediation effect, significance was satisfied when *p* < 0.05 [86].

## 3. Results

### 3.1. Descriptive and Statistical Analysis

Results of the Mann–Whitney test showed that Generation Z were more willing to include cultured meat in consumption than Generation Y (*p* < 0.05; Table 1). The Mann–Whitney test also showed that Generation Z were significantly more likely to consider cultured meat healthier because of the possibility of adjusting the composition and nutrient content; they were also significantly less concerned about adverse health effects from consuming cultured meat (both *p* < 0.05; Questions 6 and 7).

### 3.2. Structural Equation Modeling

Based on the defined constructs, SEM was carried out with the aim of determining the influence of individual factors (ethics, ecology and health) on the willingness to include cultured meat in consumption.

**Figure 1:**
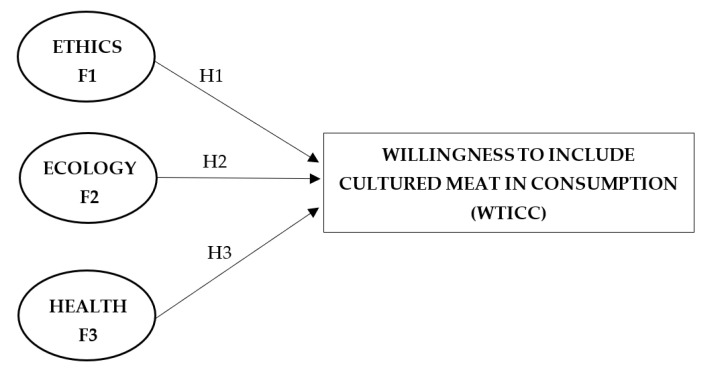
Conceptual model for structural equation modeling. Factors included were ethical and ecological awareness around cultured meat and belief in its health benefits and safety; the outcome variable was willingness to include cultured meat in consumption.

For this purpose, the following model with null and alternative hypotheses was created (Figure 1).

### 3.3. Model Representativeness

Confirmatory factor analysis of the model indicated a very good fit of the model (Table 2).

The results of SEM indicated that both ethical and ecological awareness around cultured meat, as well as belief in its health benefits and safety, had a positive effect on the willingness to include cultured meat in consumption by Generation Y and Z (Table 3).

### 3.4. Hypothesis Testing

Based on the results of the model, we concluded the following:

- There was a significant positive relationship between ethical awareness around cultured meat and willingness to include cultured meat in consumption (*p* = 0.001); the model predicted that if the level of F1 increased by 1, the level of WTICC would increase by 0.689;
- There was a significant positive relationship between ecological awareness around cultured meat and willingness to include cultured meat in consumption (*p* < 0.001); the model predicted that if the level of F2 increased by 1, the level of WTICC would increase by 0.641;
- There was a significant positive relationship between the belief in the health benefits and safety of cultured meat and willingness to include cultured meat in consumption (*p* = 0.004); the model predicted that if the level of F3 increased by 1, the level of WTICC would increase by 0.42.

**Figure 2:**
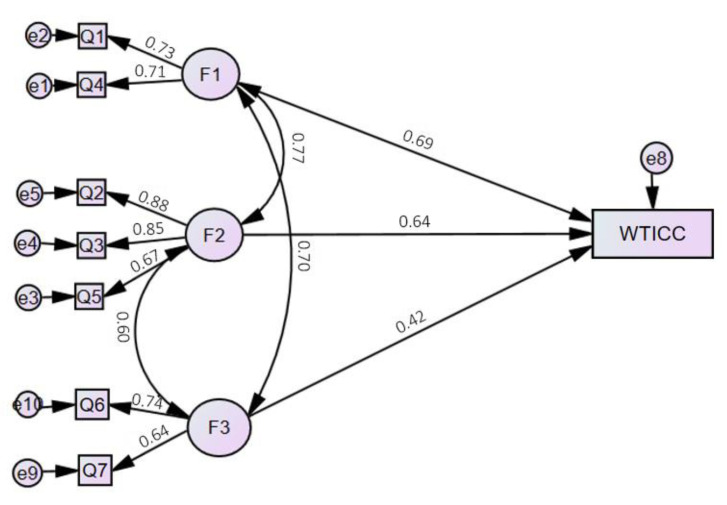
Measurement model. The effect of three factors (F) on willingness to include cultured meat in consumption. F1, ethical awareness; F2, ecological awareness; F3, belief in health benefits and safety; Q1–Q7, questions from the questionnaire; WTICC, willingness to include cultured meat in consumption.

Based on the values of the regression coefficients (0.689; 0.641; 0.42), all null hypotheses were rejected (Figure 2):

**H1_0_:**  Ethical awareness does not have a positive effect on the willingness to include cultured meat in consumption; an alternative hypothesis is accepted: H1_A_—Ethical awareness has a positive effect on the willingness to include cultured meat in consumption.

**H2_0_:**  Ecological awareness does not have a positive effect on the willingness to include cultured meat in consumption; an alternative hypothesis is accepted: H2_A_—Ecological awareness has a positive effect on the willingness to include cultured meat in consumption.

**H3_0_:**  Belief in health benefits and safety does not have a positive effect on the willingness to include cultured meat in consumption; an alternative hypothesis is accepted: H3_A_—Belief in health benefits and harmlessness has a positive effect on the willingness to include cultured meat in consumption.

## 4. Discussion

In this study, we sought to investigate the effect of ethical, ecological and health and safety factors on the willingness to consume cultured meat in people from Generations Y and Z. Our findings, along with those of recent articles [27,87], suggest that there is potential for growth in the adoption of cultured meat if the factors influencing consumer willingness are considered. We found that individuals from Generation Z were more willing to incorporate cultured meat into their diet than those from Generation Y. Although no statistically significant differences were found in the areas of ethics and ecology, significant differences were observed in the health domain. Specifically, Generation Z considered cultured meat to be healthier, because of the possibility of adjusting the composition and nutrient content of cultured meat; in addition, Generation Z had fewer concerns about the consequences of consuming cultured meat on human health than Generation Y. These findings are consistent with those of previous research and extend current knowledge on attitudes towards alternative food sources among different generations.

Compared with Generation Y, Generation Z considered cultured meat to be healthier because of the possibility of adjusting the composition and nutrient content, which could be related to their willingness to embrace new technologies to achieve a better and healthier life. This may be attributable to Generation Z growing up in an era of rapid technological innovation and digitization. Technology has become a natural part of life for this generation, leading to greater comfort and confidence in using it despite the degree of risk associated with it: one manifestation of this is the willingness to accept cultured meat as part of their diet. Although this is a relatively new and evolving concept, Generation Z are willing to experiment with this new food source in the hope of a healthier lifestyle. This outlook may have a significant impact on the adoption and diffusion of new technologies and innovations such as cultured meat in the future.

SEM showed that ethical and ecological awareness around cultured meat and belief in its health benefits and safety had a positive influence on the willingness to incorporate cultured meat into consumption among Generation Z. These findings resulted in the acceptance of the alternative hypotheses (H1_A_, H2_A_, and H3_A_), suggesting that these factors play a crucial role in deciding on the consumption of cultured meat.

Communication with consumers regarding these factors is key. Baum et al. (2021) [27] highlighted the importance of providing information to consumers and its impact on attitudes and purchasing evaluations. In their study, the authors found that the valence and complexity of information influence explicit attitudes and purchasing evaluations, but not implicit attitudes. This means that if consumers have more information about cultured meat and this information has a positive valence (i.e., is perceived as beneficial), and it is presented in an understandable manner (i.e., with lower complexity), consumers tend to have positive explicit attitudes and high evaluations of cultured meat. However, this information may not necessarily affect their implicit attitudes, which may be subconscious and less malleable. In addition, Leung et al. (2023) [88] emphasized the importance of psychological well-being in relation to the acceptance of cultured meat. Campaigns and marketing strategies could therefore focus on improving consumers’ sense of psychological well-being by increasing awareness of the potential benefits of cultured meat, such as its environmental and health benefits. This may increase consumers’ willingness to include cultured meat in their diets. Finally, Mancini and Antonioli (2022) [87] suggest that improving the taste, texture, and overall enjoyment of consuming cultured meat is needed to make it more appealing to a wider audience. In the context of this study, we confirmed that communicating (a) ethical benefits, (b) ecological benefits, and (c) health benefits and safety are key in determining the likelihood of cultured meat consumption. Specifically, health benefits and safety are important factors that forms a barrier for Generation Y to include cultured meat in their diet.

In summary, our findings build on those of previous studies and suggest that there is potential for increasing acceptance of cultured meat among Generation Y and Generation Z if factors influencing consumer willingness are taken into account. Future research could explore additional factors that may affect acceptance of cultured meat among other demographic groups and further enhance our understanding of how to promote wider acceptance of cultured meat as a sustainable protein source. This is an important area for further investigation and could provide valuable insights for promoting the adoption of cultured meat.

### 4.1. Theoretical Implications

This study provides theoretical implications for understanding the willingness to incorporate cultured meat into consumption across different generations. The findings highlight the importance of ethical awareness and ecological awareness around cultured meat and belief in its health benefits and safety in shaping attitudes towards alternative food sources. This extends the current literature on attitudes and willingness to consume cultured meat.

### 4.2. Practical Implications

The results of this study have practical implications for producers and promoters of cultured meat. To successfully introduce cultured meat into the market and increase its consumption, it is essential to understand the factors influencing the willingness of different generations to include this type of food in their diet. Producers should focus on communicating the ethical, ecological, and health advantages of cultured meat and address any concerns related to its consumption, particularly among Generation Y.

### 4.3. Future Research

A number of social and economic characteristics affect the selection and consumption of food. This research identified differences in willingness to include cultured meat in consumption in terms of year of birth (generation). For a wider acceptance of cultured meat in the diet, a deeper understanding of the individual determinants that influence the willingness to try and consume the product is required. Further research could focus on other determinants that influence the selection of food, such as education, income, family status, social environment, and health status, and how these vary between countries.

## 5. Conclusions

Overall, our findings support the results of previous studies, and suggest there is potential for increased adoption of cultured meat if various factors influencing consumer willingness are considered. SEM showed that ethical and environmental awareness around cultured meat and belief in its health benefits and safety have a positive effect on the willingness to include cultured meat in consumption among Generation Y and Generation Z. The results of the Mann–Whitney U-test show that Generation Z are more willing to include cultured meat in their consumption than Generation Y, because Generation Z are less concerned about the potential health consequences of consuming cultured meat than Generation Y. Specifically, no statistically significant differences were found in the areas of ethics and ecology, although significant differences were observed in the area of health: Generation Z viewed cultured meat as potentially healthier because of the possibility of modifying its composition and nutrient content, and they were less concerned about the human health consequences of consuming cultured meat than Generation Y.

Upon comparison of the results, it is evident that ethical, ecological, and health factors significantly influence the willingness of both Generation Y and Generation Z to incorporate cultured meat into their consumption patterns. However, Generation Y exhibit a more profound level of mistrust regarding the health implications of consuming cultured meat in comparison to Generation Z. This mistrust can be identified as a barrier to the successful implementation of cultured meat in the market. Therefore, it is imperative to address this issue effectively and ensure its communication specifically tailored to Generation Y.

## Figures and Tables

**Table 1:** Differences between Generation Y and Generation Z regarding willingness to include cultured meat in consumption.

Number *	Abbreviated Question	Motivation	Gen Y	Gen Z	Mann–Whitney Test
Q1	The meat industry presents significant ethical problems	Ethics	3.42	3.37	0.312
Q2	The meat industry presents significant ecological problems	Ecology	3.6	3.5	0.442
Q3	Meat alternatives represent a potential method by which to improve ecological problems	Ecology	3.4	3.4	0.559
Q4	Cultured meat will be more ethical than conventional meat	Ethics	3.5	3.5	0.810
Q5	Cultured meat will be more ecological than conventional meat	Ecology	3.2	3.3	0.938
Q6	Cultured meat will be healthier because of the possibility of adjusting its composition and nutrient content	Health	3.7	3.9	0.040 *
Q7	Consumption of cultured meat may affect human health	Health	3.1	2.9	0.029 *
WTICC	Willingness to include cultured meat in consumption		3.88	4.12	0.014 *

* Q1–Q7, questions from the questionnaire; WTICC, willingness to include cultured meat in consumption.

**Table 2:** Model Fit indices.

Index	Required Value	Model Value	Estimate (r)
GFI	>0.9	0.977	Yes
RMSEA	>0.08	0.067	Yes
NFI	>0.9	0.988	Yes
TLI	>0.9	0.986	Yes
CFI	>0.9	0.994	Yes
IFI	>0.9	0.994	Yes
CMIN/DF	<3	2.186	Yes

CMID/DF, discrepancy divided by degree of freedom; GFI, goodness-of-fit index; RMSEA, root mean square error of approximation; NFI, normed fit index; TLI, Tucker–Lewis coefficient; CFI, comparative fit index; and IFI, incremental fit index.

**Table 3:** Results of structural equation modeling.

			Estimate (r)	S.E.	C.R.	*p*
Q4	←	F1	0.712	f.p.		
Q1	←	F1	0.728	0.099	10.073	***
Q5	←	F2	0.672	f.p.		
Q3	←	F2	0.845	0.103	12.474	***
Q2	←	F2	0.883	0.103	12.739	***
WTICC	←	F1	0.689	0.321	3.18	0.001
WTICC	←	F2	0.641	0.243	4.189	***
Q7	←	F3	0.639	f.p.		
Q6	←	F3	0.736	0.143	7.638	***
WTICC	←	F3	0.42	0.258	2.841	0.004

Q1–Q7, questions from the questionnaire; F, factor; S.E., standard error; C.R., composite reliability; WTICC, willingness to include cultured meat in consumption; *** *p* < 0.001; f.p.—fixed parameter.

## Data Availability

As this article is supported by the long-term intent of an internal grant agency, the data are available upon request from the corresponding author.
